# Book Notices

**Published:** 1876-10

**Authors:** 


### BOOK NOTICES.

The Histology and Histochemistry of Man. A treatise on the elments of composition and structure of the human body. By Heinrich Frey, Professor of Medicine in Zurich. Translated from the fourth German edition, by Arthur E. J. Barker, Surgeon to the City of Dublin Hospital, etc., etc. Aud revised by the author, with six hundred and eight illustrations on wood. New York: D. Appleton & Co., 549 and 551 Broadway. 1875. For sale by Burke, Hancock & Co., Atlanta, Ga.

This is an exceedingly valuable work, as is shown by the title page. A brief plan of the work is given by the author. He says: "Histology and Histochemistry combined, or the study of the finer structure and chemical composition of parts, constitutes the most important foundation for physiology and scientific pathclogy. The whole subject may be arranged, accord ing to our views, into three great natural divisions.

In the first will be considered *the matters of which the human and animal body generally is composed*, with their histological and (as far as inseperable from these, and that a knowledge of them is indispensable for a proper comprehension of the whole) their physiological characters. Again, in another section of the same, the organized units of the body, the "*structural or tissue-elements*," will be brought in view with their shape and composition, purposes and origin, one from another. This constitutes "General Histology and Histochemistry."

In the second division—histology in the more restricted and real meaning of the word—*the various tissues in their anatomical relations and composition*, wlll be brought under notice. Here, also, we shall consider the mode in which the form or structural elements of the first division are employed in the building up of certain tissues. It stands to reason that here also the physiological characters of the tissues and their origin must still be frequently referred to.

A third division, finally, will be devoted to the consideration of *the more minute structure of the organs and systems* of our body, or the manner in which they are put together out of different tissues. This may be termed "Topographical Histology."

A Manuel of Diet on Health and Diseases, by Thomas King Chambers, M. D., Oxon., F. R. C. P., London. Honorary Physician to H. R. H., the Prince of Wales; etc., etc. Philadelphia: Henry C. Lea; 1875.

On Paralysis from Brain Disease in its Common Forms, by H. Charlton Bastian, M.A., M.D., F.C.P., etc., etc.; with illustrations. New York: D. Appleton & Co., 549 and 551 Broadway; 1875.

The work is divided into three parts and twenty-five chapters, each of which is devoted to the consideration of the following subjects:

Theories of Dietetics; Choice of Food; Preparation of Food; Digestion; Nutrition; Regimen of Infancy and Motherhood; Regimen of Childhood and Youth; Commercial Life; Literary and Professional Life; Noxious Trades; Athletic Training; Hints for Healthy Travelers; Effects of Climate; Starvation, Poverty and Fasting; Decline of Life; Alcohol; Dulelics and Regimen of Acute Fevers; Diet and Regimen of Certain Other Inflammatory States; Diet and Regimen of Weak Digestion; Gout and Rheumatism; Gravel, Stone, Albuminuria and Diabetes; Deficient Evacuation; Nerve Disorders; Scrofula, Rickets and Consumption; Disease of Heart and Arteries.

This gives the reader an idea of the rich contents of the book. It is practical, and will be of great usefulness to households, to say nothing of physicians. There is a fund of valuable information to be found in its pages, and no physician can afford to be without it.

These lectures appeared in the London *Lancet*, and were delivered in University College Hospital. The subjects are ably discussed, and cases given illustrative of the various brain diseases resulting in paralysis, and the treatment employed. It is a work that will serve the busy practitioner a good purpose in actual practice, while a study of its pages will prepare him all the better for cases of brain disease which he may be called upon to treat. The matter is given in a clear and practical manner, and affords pleasant reading independent of the great importance of the subjects treated by the author.

Transactions of the Medical Association of Alabama—A State Board of Health. 29th Session, 1876.

This is quite a large volume, devoted to the proceedings of the Association, and two lengthy papers—the first a prize essay on the pathology and treatment of Bright's disease, by Dr. H. D. Schmidt, of Mobile; a very able and exhaustive paper; the other on "Hemorrhogic Malarial fever," by Dr. R. D. Webb, of Livingston, Ala. Also, a very practical and ably written paper. These papers are too valuable to be entombed in a volume of transactions, where few of the profession will ever see them. Dr. E. D. McDaniel is President, and Dr. B. H. Riggs is Secretary of the Association.

Transactions of the Medical and Chirugical Faculty of Maryland, 78th Annual Session, April, 1876.

This is a well filled volume, containing several papers of interest.

Transactions of the Medical Association of the State of Missouri, at its 10th Annual Session, St. Louis, April, 1876.

This volume also presents papers of value.

Proceedings of the Medical Society of the County of Kings, Brooklyn, New York, September, 1876.

Some papers of practical importance appear in these proceedings, one of which we publish in the present uumber of the RECORD.

The following pamphlets have been received : An Address on on some of the leading public health questions, and some remarks of the extent of swamp lands in the United States, etc., by J. M. Toner.

A clinical lecture on the treatment of incipient stricture, by Otis's operation, by Mr. Berkely Hill, with explanatory remarks by Tressenden N. Otis, M.D.

Report of the Committee on Medical Education, made to Medical Society of California, by Jos. F. Montgomery, M. D.

A Clinical Lecture, on the use of plastic dressing in fractures of lower extremity, by David Yandell, M. D.

On Stricture of the Male Urethra, its radical cure, by Fressenden N. Otis, M. D.

A series of American Clinical Lectures: edited by E. C. Seguin, M. D. Vol. II, No. 6—Whole No. 18.

The Modern Methods of Examining the Upper Air-passage, by George M. Lefferts, M. D.

These series of clinical lectures are of great practical importance, and should have an extensive reading by the profession. They are published by G. P. Putnam's Sons, 182 Fifth Avenue, New York. The first volume is now ready. Price $4.00.

